# Effect of pomegranate flower extract on cisplatin-induced nephrotoxicity in rats

**DOI:** 10.12860/jnp.2014.26

**Published:** 2014-10-01

**Authors:** Fatemeh Motamedi, Mehdi Nematbakhsh, Ramesh Monajemi, Zahra Pezeshki, Ardeshir Talebi, Behzad Zolfaghari, Azam Mansoori, Shadan Saberi, Aghdas Dehghani, Farzaneh Ashrafi

**Affiliations:** ^1^Water & Electrolytes Research Center, Isfahan University of Medical Sciences, Isfahan, Iran; ^2^Department of Biology, Falavarjan Branch Islamic Azad University, Isfahan, Iran; ^3^Department of Physiology, Isfahan University of Medical Sciences, Isfahan, Iran; ^4^Institute of Basic & Applied Sciences Research, Isfahan, Iran; ^5^Department of Clinical Pathology, Isfahan University of Medical Sciences, Isfahan, Iran; ^6^Department of Pharmacognosy, Isfahan University of Medical Sciences, Isfahan, Iran; ^7^Department of Internal Medicine, Isfahan University of Medical Sciences, Isfahan, Iran

**Keywords:** Pomegranate flower extract, Cisplatin, Nephrotoxicity

## Abstract

*Background: * Chemotherapy with cisplatin (CP) is accompanied with nephrotoxicity.

*Objectives:* In the current study, pomegranate flower extract (PFE) has been evaluated as an antioxidant agent against CP-induced-renal toxicity.

*Materials and Methods:* Thirty two male Wistar rats were divided into five groups (6-8 in each group). The animals in groups 1 to 3 received PFE (25 mg/kg), PFE (50 mg/kg), and placebo (saline), respectively for 9 days, and onset of the day 3, they also received CP (2.5 mg/kg/day). Groups 4 and 5 were treated with PFE (25 and 50 mg/kg/day) for 9 days. Finally, the animals were sacrificed at day 9 after collecting blood samples. Kidneys were removed, weighted, and underwent histopathological investigation.

*Results: * The mean serum level of creatinine in group 3 (treated with CP and placebo) increased significantly (p<0.05), but the value decreased significantly (p<0.05) in group 1. Kidney weight in group 1 was lower than KW in groups 2 and 3, however it was significant when compared with group 2 (p<0.05). The serum nitrite level in group 2 was non-significantly lower than that in other groups, and no significant changes were observed in serum levels of malondialdehyde (MDA). Tissue level of nitrite was significantly decreased in the positive control and high dose of PFE plus CP-treated groups (p<0.05). Among CP-treated groups, low dose of PFE significantly improved kidney nitrite level (p<0.05). The results from histopathological staining indicated less tissue damage in group 1 when compared with group 3.

*Conclusions: * It seems that low dose of PFE plays a protective role against CP-induced renal toxicity in rats.

**Implication for health policy/practice/research/medical education:**

The low dose of pomegranate flower extract (25 mg/kg) provided protective effects and ameliorated cisplatin-induced nephrotoxicity through its antioxidant effects. Therefore, co-administration of pomegranate flower extract with cisplatin might be beneficial for these patients.

## 1. Background

Cisplatin (Cis-diamminedichloroplatinum, CP) has been successfully used as a chemotherapeutic agent in the treatment of solid tumors (1). Despite its potent anti-neoplastic action, it has many side effects such as hepatotoxicity, ototoxicity, neurotoxicity, gastrotoxicity, myelosuppression, and allergic reactions (2). The major side effect of CP is nephrotoxicity. Nephrotoxicity is a result of proximal tubular cell injury which occurs with high prevalence in the initial days of CP chemotherapy. The mechanism of CP-induced nephrotoxicity might be related to oxidative stress, apoptosis or an inflammatory response (2). CP-induced oxidative stress and inflammatory response in the kidney may partially be prevented by several chemical and natural compounds such as L-arginine, losartan and erythropoietin (3-6). Oxidative stress is responsible for a variety of degenerative processes in some diseases and is initiated by free radicals which cause protein and DNA damage along with lipid peroxidation (7,8). Most herbs have antioxidant properties. *Morus alba* leaves have been reported to be effective against CP-induced nephrotoxicity (6). Also, the olive leaves have chemical content and antioxidant properties that remove free oxygen radicals (9). Pomegranate parts including leaf, bark, root, fruit, juice and seed contain ingredients that can be effective as antimicrobial and antioxidant agents (10). Pomegranate fruit and juice have anti-fungal (10) and anti-inflammatory properties (11) and reduce blood pressure (12). The major phenolic composition of pomegranate flower extract (PFE) is gallic acid, which has protective effect in kidney disturbances (13). The aqueous extract of pomegranate was investigated in gentamicin-induced nephrotoxicity model and its protective effects was reported (14).

## 2. Objectives

Considering the antioxidant properties of pomegranate, we examined the effects of PFE on CP-induced nephrotoxicity in the current study.

## 3. Materials and Methods

### 3. 1. Animals 

This is an experimental study in which 32 adult male (170-200 g) Wistar rats (Animal Center, Isfahan University of Medical Sciences, Isfahan, Iran) were used. The rats were housed at the temperature of 23-25 °C and had free access to water and rat chow. The rats were acclimatized to this diet for at least one week prior to the experiment. The experimental procedures were in advance approved by the Isfahan University of Medical Sciences Ethics Committee.

### 3.2. Drugs

CP was purchased from EBEWE Pharma Ges.m.b.H (Unterach, Austria), and ketamine HCl was obtained from Rotexmedica (Trittau, Germany).

### 3.3. PFE preparation

Five hundred grams of pomegranate flower was provided and powdered. Hydroalcoholic extract was prepared by ethanol: water (70:30) mixture using percolation method. Hydroalcoholic extract was concentrated and dried to obtain 123 g pure powder.

### 3.4. Measurement of extract total phenolic content

Total phenolic content of the extract was assessed by the Folin-Ciocalteu method. Briefly, 20 µl extract 85% plus 1.58 ml deionized water and 100 µl Folin-Ciocalteu reagents were mixed. After 30 s, 30 µl Na_2_CO_3_ was added to the mixture. Then, the mixture was incubated at 20 °C for 2 h. Finally, the absorbance was read in 765 nm.

### 3.5. PFE analysis

PFE analysis indicated that the major phenolic content of this extract is gallic acid (9.98%).

### 3.6. Experimental protocol

The animals were randomly assigned to five groups and were treated as follows: Group 1 (n= 6) received PFE (25 mg/kg/day) for 9 consecutive days and from day 3 CP was added to the treatment (2.5 mg/kg/day). Group 2 (n= 6) had treatment the same as group 1with PFE at the dose of 50 mg/kg/day. Group 3, as the positive control group, (n= 8) was treated similar to group 1, however, received saline instead of PFE.

Groups 4 (n= 6) and 5 (n= 6) as negative control groups received 25 and 50 mg/kg/day of PFE alone for 9 consecutive days, respectively. Animal body weight was recorded on a daily basis. At the end of the experiment, the animals were anesthetized with ketamine (75 mg/kg, ip), blood samples were collected by heart puncture and the animals were sacrificed. The kidneys were removed immediately and weighed. Left kidney was fixed in 10% neutral formalin solution and right kidney was homogenized in 2 ml of saline for the measurements. Two additional groups of animals were also treated in this study. Group A (n= 6) received treatment the same as group 1 except PFE (100 mg/kg/day) and group B (n= 6) received 100 mg/kg/day PFE alone. The mortality rates in these two groups were above 70%. Therefore, their results were not considered in data analysis. The reason of this high mortality rate seems to be related to high dose of drug.

### 3.7. Measurements

Serum level of creatinine (Cr) was determined using quantitative diagnostic kits (Pars Azmoon, Iran). The serum and kidney levels of nitrite (stable NO metabolite) were measured using a colorimetric assay kit (Promega Corporation, USA) based on the Griess reaction. Serum and kidney levels of malondialdehyde (MDA) were quantified according to the manual method. Briefly, 500 µl of the sample was mixed with 1000 µl of 10% trichloroacetic acid (TCA). The mixture was centrifuged at 2000 g for 10 min; 500 µl of the supernatant was added to 500 µl of 0.67% thiobarbituric acid (TBA). Then, the solution was incubated for 10 min in warm water bath at the temperature of 100 °C. After cooling, the absorbance was measured at the wavelength of 532 nm.

### 3.8. Histopathological procedures

Left kidney was fixed in 10% neutral formalin solution and embedded in paraffin. Tissue sections were stained with hematoxylin and eosin method. The kidney tissue damage score (KTDS) was determined by a pathologist who was blind to the study. The samples were scored from 1 to 4, while score 0 was assigned to normal kidney tissue without damage. Parameters of tubular damage included tubular dilation, tubular cells swelling and necrosis, tubular casts, and intraluminal cell debris.

### 3.9. Statistical analysis

Data was expressed as mean ± SD and median, minimum and maximum. The levels of Cr, MDA, and nitrite, as well as body weight and kidney weight (KW) were analyzed by the one-way ANOVA followed by the Tukey’s test using SPSS 16 Software. The tissue damage scores were compared by the Kruskal-Wallis or Mann-Whitney tests.

## 4. Results

### 4.1. Serum level of Cr

**Figure 1:**
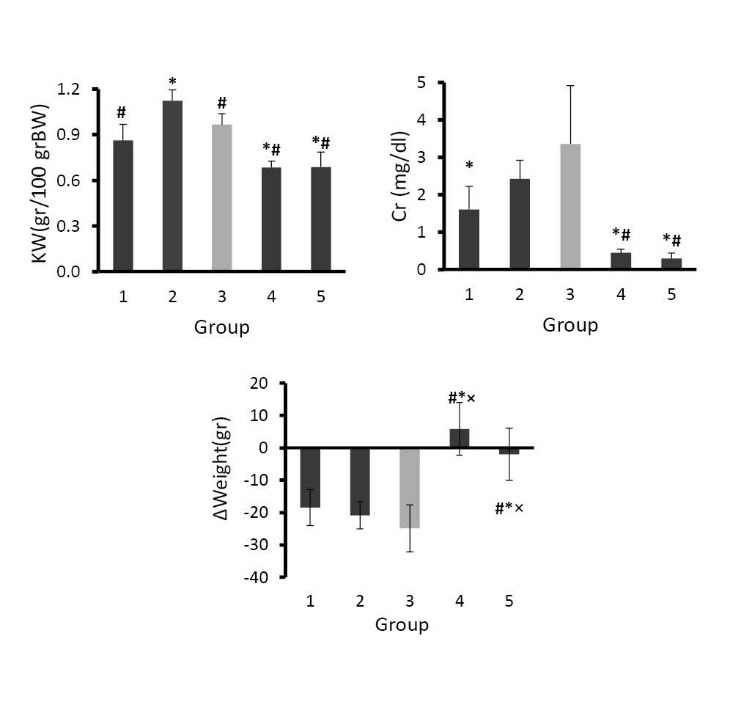
The data for serum level of creatinine (Cr, above right), total kidney weight (g)/100 (g) body weight (KW, above left), delta weight (∆W, weight difference, below), in five experimental groups (see text for group details). One-way ANOVA was applied for analyses. (*), (#) and (×) indicate significant difference (p<0.05) when compared with the positive control (group 3), group 2, and group 1, respectively.

The mean serum level of Cr in group 3 (treated with CP and placebo) increased significantly when compared with groups 4 and 5 (p<0.05). This finding verified CP-induced nephrotoxicity. The low dose of PFE when accompanied with CP (group 1) significantly decreased the serum level of Cr (p<0.05), while such observation was not detected in group 2 (Figure 1). Statistical analysis also indicated that no difference was observed between serum Cr levels in groups 1, 4, and 5. This findings indicated that low dose of PFE potentially could reduce the Cr level increased by CP administration.

### 4.2. Body weight, kidney weight, and KTDS

**Figure 2:**
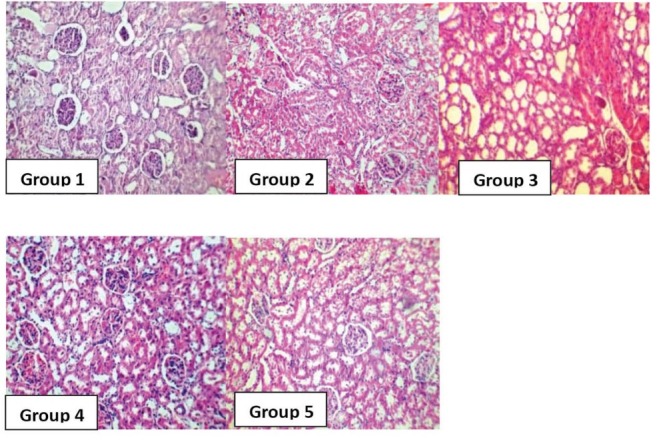
Micrographs of kidney tissue damage score in five experimental groups (see text for groups details). No damage was seen in groups 4 and 5. The tissue damage in group 1 is less than that in groups 2 and 3.

Both KW and KTDS increased significantly in the positive control group (group 3) when compared with the negative control groups (p<0.05). In addition, significant increase of KW was seen in group 2 compared with the positive control group (p<0.05). In all CP-treated animals, the weight loss were significant, and KTDS were also significantly higher when compared with the negative control groups (p<0.05;Table 2). However, the intensity of weight loss and KTDS in group 1 was lower than those in other CP-treated groups (Figure 1; Table 2). The images of kidney tissue samples are demonstrated in Figure 2.

**Table 2:** The minimum and maximum range, median and mean± SD of kidney damage score in five experimental groups

**Group**	**Minimum**	**Maximum**	**Median**	**Mean**
CP +PFE 25 (mg/kg)	2	3	2	2.2±0.45
CP + PFE 50 (mg/kg)	2	3	2.5	2.5±0.55
CP	2	3	2	2.38±0.52
PFE 25 (mg/kg)	0	1	0	0.25±0.5^#*×^
PFE 50 (mg/kg)	0	0	0	0±0.00^#*×^

(*), (#) and (×) indicate significant difference (P<0.05) when compared with the positive control (group 3), group 2, and group 1, respectively.

### 4.3. Serum and kidney nitrite and MDA

The serum nitrite level in group 2 was lower than that in other groups. However, no significant changes were observed in serum levels of nitrite and MDA and kidney tissue MDA in all experimental groups. Tissue levels of nitrite significantly decreased in the positive control and high dose of PFE plus CP-treated groups (p<0.05). Among CP-treated groups, low dose of PFE significantly improved kidney nitrite level (p<0.05; Table 1).

**Table 1:** Serum and kidney level of malondialdehyde (MDA) and nitrite (NO)

**Group**	**Serum nitrite (µmole/L)**	**Kidney nitrite (µmole/g tissue)**	**Serum MDA (µmole/L)**	**Kidney MDA** **(nmole/g tissue)**
CP +PFE 25 (mg/kg)	16.60 ± 4.98	0.23 ± 0.07^*#^	12.07 ± 8.65	4.53 ± 1.64
CP + PFE 50 (mg/kg)	8.23 ± 2.75	0.13 ±0.03	15.40 ± 8.21	3.46 ± 2.25
CP	18.58 ± 12.08	0.12 ± 0.05	11.58 ± 10.59	4.40± 0.74
PFE 25 (mg/kg)	17.55 ± 6.99	0.23 ± 0.11^*^	11.47 ± 9.21	3.52 ± 1.77
PFE 50 (mg/kg)	14.83 ± 3.68	0.26 ± 0.08^*#^	14.11 ± 5.30	5.41 ± 0.91

(*) and (#) indicate significant difference (p<0.05) when compared with the positive control and the PFE 50+CP groups, respectively

## 5. Discussion

The aim of this study was to evaluate the protective effect of PFE against CP-induced renal toxicity. We found that low dose of PFE could ameliorate CP-induced kidney toxicity. CP injection induces kidney tissue damage in animals (15,16), and also increases the serum levels of BUN and Cr, and KW (17,18). CP also affects the serum level of MDA (19). Body weight loss in CP-treated group occurs following the gastrointestinal disturbance (20,21). It has been reported that administration of high dose of pomegranate fruit extract decreased body weight (22). This possibly indicates that tannins in pomegranate juice interacts with proteins, inhibit protein digestion (23). In contrast, the KW gain was observed in CP-treated groups. It is reported that KW increased in CP-treated animal, and it has a direct relationship with intensity of tissue damage (6). The low dose of PFE plus CP led to reduction in KW and KTDS whereas high dose of PFE with CP increased KW. Possibly the protective effect of PFE in low dose was attributed to its antioxidant properties (24). CP also decreased the level of nitrite in kidney tissue. NO, as a marker of endothelial function, plays a critical role in endothelial and NO is synthesized from the amino acid L-arginine by the endothelial NO synthase (eNOS) in endothelium. CP can disturb endothelium and endothelial function followed by reducing NO (3,25). Accordingly, the increase of kidney nitrite level by low dose of PFE has improved kidney NO to protect the kidney from endothelial dysfunction. Administration of PFE in low dose also ameliorated KTDS induced by CP. It has been shown that hydroalcoholic extract of pomegranate flowers can potentially improve recovery of kidney function that may be related to NO-dependent signaling pathway (26). We did not observed protective role of PFE in 50 mg/kg against CP-induced toxicity in the current animal model. It seems that it was related to the antioxidant dose, because high doses of some antioxidants do not have a protective effect, and can exacerbate tissue damage (27,28), while, other antioxidants such as Morus alba L. leaf extracts had protective role against CP induced nephrotoxicty (6). Although we did not observed significant difference in serum MDA levels between the groups, but according to other parameters such as Cr, KW and KTDS, the PFE in 25 mg/kg act as antioxidant against CP-induced nephrotoxicity. Therefore, similar study to investigate PFE in doses lower than 25 mg/kg is suggested.

## 6. Conclusions

The low dose of PFE (25 mg/kg) showed protective effects and ameliorated CP-induced nephrotoxicity through its antioxidant effects.

## Authors’ contributions

All authors wrote the manuscript equally.

## Conflict of interests

The authors declared no competing interests.

## Funding/Support

This research was supported by Isfahan University of Medical Sciences.
